# Cardiac remodelling and clinical outcomes after mitral edge-to-edge repair with the PASCAL® system

**DOI:** 10.1093/ehjimp/qyag106

**Published:** 2026-06-12

**Authors:** Mareike Bladt, Marius Keller, Meinrad Gawaz, Juergen Schreieck, Andreas Goldschmied, Makoto Amaki, Takashi Matsumoto, Izumo Masaki, Shingo Kuwata, Shunsuke Kubo, Kentaro Hayashida, Firas Zahr, Felix Kreidel, Fabien Praz, Didier Tchétché, Harry Magunia, Monika Zdanyte, Tobias Geisler

**Affiliations:** Department of Cardiology and Angiology, University Hospital Tübingen, Otfried-Müller-Straße 10, Tübingen 72076, Germany; Department of Anaesthesiology and Intensive Care Medicine, University Hospital Tübingen, Hoppe-Seyler-Street 3, Tübingen 72076, Germany; Department of Cardiology and Angiology, University Hospital Tübingen, Otfried-Müller-Straße 10, Tübingen 72076, Germany; Department of Cardiology, Medius Kliniken gGmbH medius Klinik, Ostfildern-Ruit, Germany; Department of Cardiology and Angiology, University Hospital Tübingen, Otfried-Müller-Straße 10, Tübingen 72076, Germany; Department of Heart Failure and Transplant, Division of Heart Failure, National Cerebral and Cardiovascular Center, 6-1 Kishibeshinmachi Suita, Osaka 564-8565, Japan; Department of Cardiology and Cath Laboratories, Shonan Kamakura General Hospital, Kamakura, Kangawa, Japan; Division of Cardiology, Department of Internal Medicine, St.Marianna University School of Medicine, Kawasaki, Japan; Division of Cardiology, Department of Internal Medicine, St.Marianna University School of Medicine, Kawasaki, Japan; Department of Cardiology, Kurashiki Central Hospital, Kurashiki, Japan; Department of Cardiology, Keio University School of Medicine, 35 Shinanomachi, Shinjyuku-ku, Tokyo 160-8582, Japan; Division of Cardiovascular Medicine, Knight Cardiovascular Institute, Oregon Health & Science University, Portland, OR, USA; Department of Internal Medicine III, Cardiology and Critical Care, University Hospital Schleswig-Holstein, Campus Kiel, Arnold-Heller-Straße 3, Kiel 24105, Germany; Department of Cardiology, Inselspital, Bern University Hospital, Freiburgstrasse 18, Bern 3010, Switzerland; Department of Interventional Cardiology, Clinique Pasteur, Toulouse, France; Department of Anaesthesiology and Intensive Care Medicine, University Hospital Tübingen, Hoppe-Seyler-Street 3, Tübingen 72076, Germany; Department of Cardiology and Angiology, University Hospital Tübingen, Otfried-Müller-Straße 10, Tübingen 72076, Germany; Department of Cardiology and Angiology, University Hospital Tübingen, Otfried-Müller-Straße 10, Tübingen 72076, Germany

**Keywords:** mitral regurgitation, transcatheter edge-to-edge repair, haemodynamic changes, myocardial remodelling, PASCAL system

## Abstract

**Aims:**

Chronic mitral regurgitation (MR) causes haemodynamic and myocardial changes leading to increased left atrial volume and left ventricular preload and finally eccentric hypertrophy of the left ventricle. The extent to which mitral valve transcatheter edge-to-edge repair induced haemodynamic improvements translates into reverse remodelling and clinical benefit remains to be clarified. This project aimed to investigate periprocedural haemodynamic changes during mitral valve transcatheter edge-to-edge repair, their effects on reverse cardiac remodelling, and the associated effects on heart failure-related hospitalization and all-cause mortality.

**Methods and results:**

In this retrospective study, 130 patients with severe symptomatic MR underwent transcatheter edge-to-edge repair. Haemodynamic parameters were assessed pre- and immediately post-procedure, and echocardiography was performed at 6–12 months (median 188 days, IQR 26). Clinical follow-up included 1-year heart failure hospitalization and all-cause mortality. Short-term haemodynamic data were available in 86 patients and long-term echocardiographic follow-up in 73, with incomplete datasets excluded from the respective analyses. Mitral valve transcatheter edge-to-edge repair significantly improved cardiac index (+11.37%, *P* < 0.01) and reduced left atrial pressures (−36.97%, *P* < 0.01). Echocardiography demonstrated reduced left ventricular end-diastolic diameter (−2.8 mm, −4.0%, *P* = 0.004) and improved right ventricular function (+5.8%, *P* = 0.019) at follow-up. Patients with improved left ventricular global longitudinal strain had lower mortality and hospitalization rates.

**Conclusion:**

Mitral valve transcatheter edge-to-edge repair with the PASCAL® system is linked with immediate haemodynamic improvements that may be associated with reverse cardiac remodelling and improved mid-term clinical outcomes. Monitoring acute haemodynamic effects may help predict positive remodelling and outcome effects.

## Graphical Abstract

**Graphical Abstract:**
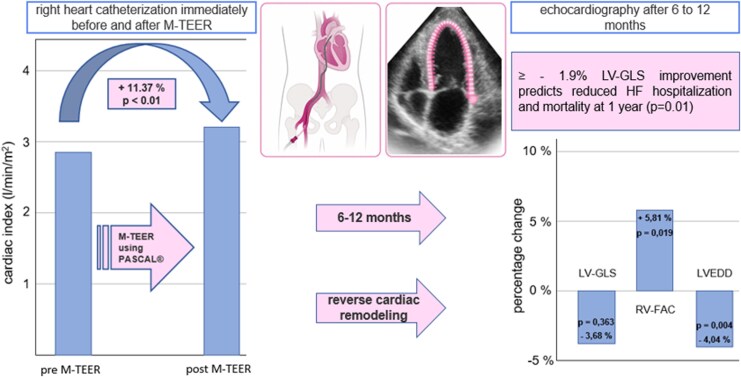
The left bar chart shows the increase in cardiac index after M-TEER using the PASCAL® system, measured by right heart catheterization (pre vs. post; +11.37%, *P* < 0.01). Arrows indicate reverse cardiac remodelling over 6–12 months. The right chart presents follow-up echocardiographic changes: LV-GLS (−3.68%, *P* = 0.363), RV-FAC (+5.81%, *P* = 0.019), and LVEDD (−4.44%, *P* = 0.004). Improved LV-GLS (≥−1.9%) was associated with reduced mortality (*P* = 0.046) and heart failure re-hospitalization (*P* = 0.023). Abbreviations: M-TEER, mitral valve transcatheter edge-to-edge repair; LV-GLS, left ventricular global longitudinal strain; RV-FAC, right ventricular fractional area change; LVEDD, left ventricular end-diastolic diameter.

## Introduction

Chronic mitral regurgitation (MR) is the most prevalent valvular heart disease in Europe.^1–3^ Patients may remain asymptomatic for extended periods due to compensatory mechanisms. MR leads to left ventricular (LV) volume overload, resulting in eccentric hypertrophy as the heart adapts to maintain adequate stroke volume, a process explained by Laplace’s law, which relates wall stress to chamber size and pressure. As the disease progresses, continued LV enlargement increases afterload and impairs contractility, ultimately reducing ejection fraction and leading to symptoms such as dyspnoea and pulmonary oedema.^4^

In addition, volume overload of the left ventricle and atrium often leads to predominantly post-capillary pulmonary hypertension, resulting from increased filling pressures and passive backflow. Pulmonary hypertension is present in 60–70% of patients with severe symptomatic mitral valve disease and is associated with a poorer prognosis.^5,6^ Left ventricular global longitudinal strain (LV-GLS), a speckle-tracking echocardiographic parameter, provides a more sensitive measure of LV damage and myocardial fibrosis than left ventricular ejection fraction (LVEF), particularly in patients with non-ischaemic cardiomyopathy and severe secondary MR.^7^ LV-GLS is independently associated with increased risk of all-cause mortality in both primary and secondary MR.^8,9^ Furthermore, in the COAPT trial, patients who demonstrated improvement in LV-GLS from baseline to 6 months after mitral valve transcatheter edge-to-edge repair (M-TEER) experienced the greatest survival benefit.^10^

M-TEER is an established minimally invasive procedure used to treat primary and secondary mitral valve regurgitation in patients who are not candidates for surgery. In selected patients with secondary MR and heart failure, M-TEER has been shown to reduce all-cause mortality and lower the rate of hospitalization for heart failure compared to medical therapy alone.^11^

Previous studies have reported that mitral valve repair with the MitraClip® system leads to favourable post-procedural haemodynamic changes, including increased stroke volume, higher cardiac output, and improved loading conditions.^12^ Additionally, improvements in right ventricular function and reductions in pulmonary artery pressure have been observed following the procedure.^13,14^ Echocardiographic data also demonstrate reduced LV dilatation and increased contractility after transcatheter mitral valve repair, particularly in patients with a LVEF >40%.^15^

The aim of this study was to systematically investigate periprocedural haemodynamic changes during M-TEER using the PASCAL® system, and to assess their impact on myocardial remodelling, clinical outcomes (including heart failure-associated hospitalization and all-cause mortality), and implant durability at 6–12 months follow-up.

## Methods

### Study design and patients

**Figure 1:**
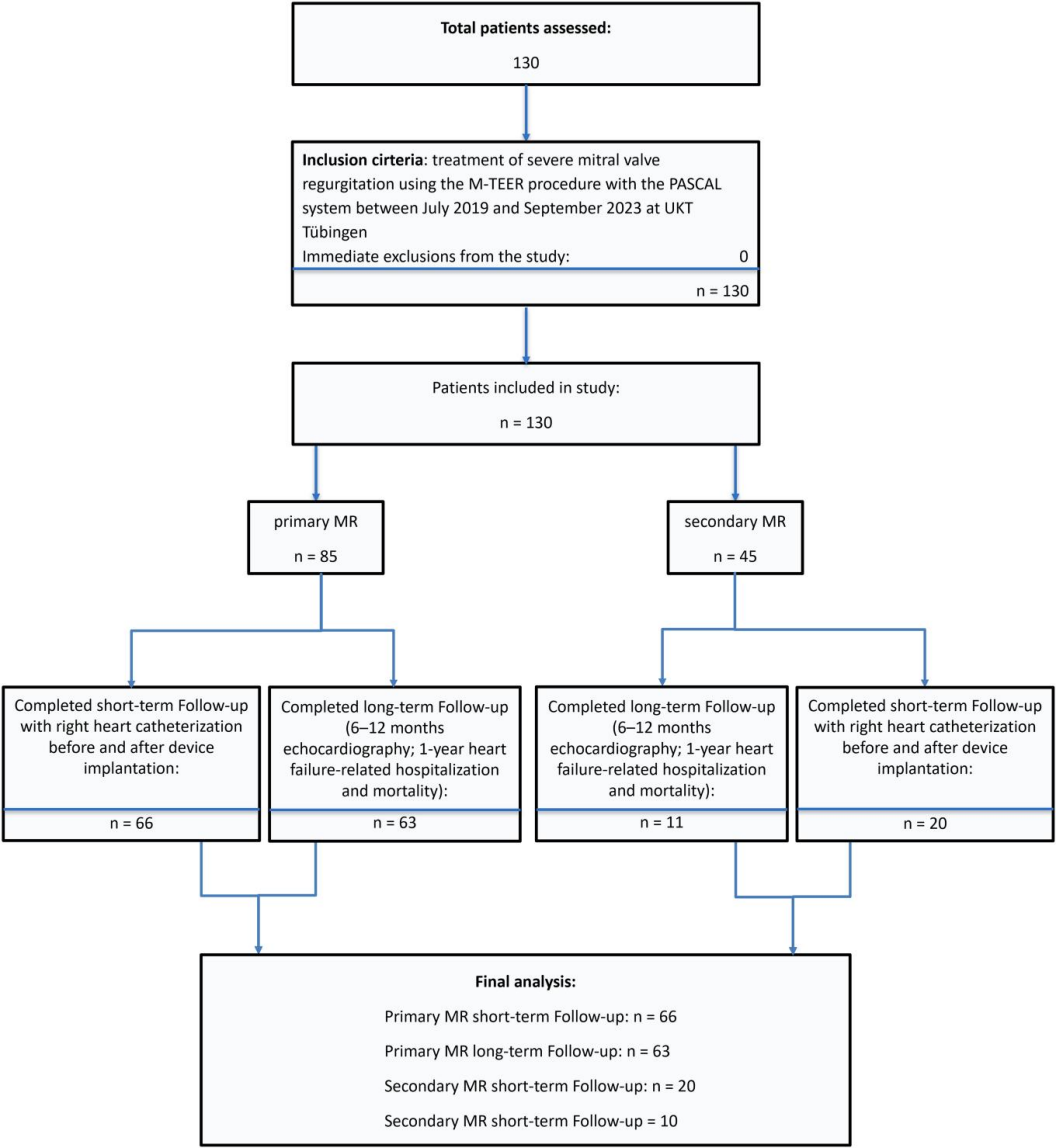
Patient flow chart. The figure shows the identification, inclusion, and exclusion of participants, as well as the final analysis cohort.

This retrospective, single-centre study included 130 consecutive patients with severe, symptomatic primary or functional MR, who underwent the M-TEER procedure with the PASCAL system between July 2019 and September 2023. The suitability for each patient for the M-TEER procedure was evaluated by a local Heart Team in accordance with the latest guidelines.^3^ The M-TEER procedure was performed under conscious sedation using propofol and midazolam.^16^ The vast majority of patients (96.2%) was breathing spontaneously. Only 3.8% were intubated and on mechanical ventilation at the time of the procedure. Intraprocedural haemodynamic parameters were determined using right heart catheterization before and immediately after device implantation. These measurements were performed as part of the standard procedure, under similar levels of sedation, blood pressure, and heart rate. Transthoracic echocardiography was performed at baseline before M-TEER, and at the 6–12-month follow-up. The median number of days between M-TEER and follow-up echocardiography was 188, with an interquartile range of 26 days. Short-term haemodynamic data were available in 86 patients and long-term echocardiographic follow-up in 74, with incomplete datasets excluded from the respective analyses. The follow-up period for all-cause mortality and hospitalizations due to heart failure was set at a fixed 12-month period for all patients. In cases where, due to the retrospective nature of the data collection, no follow-up data were available before the end of the 12-month period and could not be determined retrospectively, patients were censored at the time of the last available contact. The process of patient inclusion to follow-up is further illustrated in *Figure 1*. The study was approved by the institutional ethics committee (238/2018BO2) and complies with the Declaration of Helsinki and the good clinical practice guidelines.^17–19^

### Haemodynamic and echocardiographic parameters

Haemodynamic parameters [left atrial pressure (LA pressure), v-wave, mean pulmonary artery pressure (mPAP), pulmonary capillary wedge pressure (PCWP), and cardiac index (CI)] were measured at the beginning of the procedure and immediately after the deployment of the final PASCAL® device. The CI was calculated using the Fick principle. The steerable guide catheter was removed from the atrial septum only after oximetry had been performed, in order to prevent a left-right shunt from increasing mixed venous oxygen saturation and falsely elevating the CI.

Before M-TEER and at the 6–12-month follow-up, the following echocardiographic parameters were collected: severity of MR based on vena contracta and/or effective regurgitant orifice area (EROA); biplane LVEF according to Simpson’s method; LV-GLS; left ventricular end-systolic and end-diastolic diameter (LVEDD); tricuspid annular plane systolic excursion (TAPSE); right ventricular fractional area change (RV-FAC); and tricuspid valve peak gradient. LV-GLS and the left ventricular end-diastolic volume (LVEDV) were post-processed using IntelliSpace Cardiovascular (Philips Medical Systems Nederland), based on two-dimensional speckle tracking imaging. LV-GLS analyses were performed by two independent, experienced study physicians. The final LV-GLS value used for analysis was calculated as the mean of both measurements. Prior to the procedure, the ratio of EROA and LVEDV was also determined. A ratio of EROA (mm^2^)/LVEDV (mL) ≥ 0.15 was defined as disproportionate MR.^20^

### Study endpoints

The primary endpoint was defined as a composite endpoint of heart failure-related hospitalization and all-cause mortality after 1 year. The secondary endpoints of our study were implant durability, defined as residual MR after 6–12 months, and the correlations between immediate post-procedural haemodynamic changes and echocardiographic parameters at follow-up (6–12 months) to assess cardiac remodelling. Furthermore, we examined the relationship between these haemodynamic and echocardiographic changes and clinical outcomes to explore the pathophysiological link between remodelling and patient prognosis after M-TEER.

### Procedural success

Success was defined according to the Mitral Valve Academic Research Consortium (MVARC) criteria, which included technical, device, procedural, and patient success.^21^ Technical success was measured directly after the intervention was completed. It was defined as patient survival, successful device implantation, and an absence of emergency re-intervention or surgery. Device success was measured twice: initially after the intervention and again at the follow-up. Device success was defined as a technical success combined with a reduction in MR of at least ≥1 degree (acceptable device success), or a reduction in MR to a degree of ≤ I+ (optimal device success). Additionally, device success was only granted if there was no mitral stenosis (mean pressure gradient <5 mmHg). Device success was a prerequisite for procedural success, which was measured at follow-up. Procedural success was only granted if there was no death, no life-threatening bleeding (defined by the MVARC Bleeding Scale^21^), acute kidney injury (including new haemodialysis), or re-surgery/re-intervention. Finally, patient success was defined as device success plus the absence of re-hospitalization for heart failure after the intervention, and an improvement in New York Heart Association (NYHA) class of at least 1 degree. Patient success was also measured at follow-up.

### Statistical analysis

All statistical analyses were performed using IBM SPSS Statistics Version 28.0. A two-sided alpha level of 0.05 was applied. Normality of continuous variables was evaluated using the Kolmogorov–Smirnov test. Non-normally distributed variables were analysed using non-parametric tests. Differences between groups were assessed with the Mann–Whitney *U* test, and correlations were examined using the Spearman correlation. Continuous, normally distributed variables are presented as the mean ± standard deviation. Comparisons between different time points for the same patient were made using a paired Student’s *t*-test. Categorical variables were assessed as counts and percentages. Correlation analysis of continuous variables was performed using the Pearson correlation coefficient. Survival curves were generated using the Kaplan–Meier analysis. Additionally, a receiver operating characteristic analysis was performed to determine the LV-GLS cut-off value that best predicts mortality. Linear regression analysis was performed to evaluate predictors of improvement in LV-GLS according to the cut-off value determined by the receiver operating characteristic analysis. The odds ratio and 95% confidence intervals are stated for the covariates. Finally, a multivariate Cox regression analysis was performed to examine the relationship between the independent variables and composite endpoint of all-cause mortality and hospitalizations for heart failure.

## Results

### Reduction of MR with M-TEER

A total of 130 patients were enrolled in this study. 65.4% of the included patients were diagnosed with primary MR, and 34.6% with secondary MR. In both groups, the proportion of patients with NYHA class ≥ III exceeded 90%. The mean LVEF in patients with primary MR was 49.6%, and the mean LVEDV was 142.6 (±52.11) mL. In patients with secondary MR, the mean LVEF was 34.3%, and the LVEDV was 188.4 (±66.82) mL. During right heart catheterization, patients with primary MR had an initial mPAP of 24.4 (±8.2) mmHg, and patients with secondary MR had an initial mPAP of 29.4 (±10.5) mmHg. The baseline characteristics are shown in *Table 1*.

**Table 1:** Patient baseline characteristics

	All (*n* = 130)	Primary MR (*n* = 85)	Secondary MR (*n* = 45)	*P*-value
Age (± SD)	79.4 (±7.4)	80. (±5.7)	78.1 (±9.8)	0.936
Male sex, *n* (%)	64 (49.2)	40 (47.1)	24 (53.3)	0.581
NYHA class ≥ III, *n* (%)	123 (95)	80 (94.2)	43 (95.5)	0.382
Intubated patients while M-TEER, *n* (%)	5 (3.8)	3 (3.5)	2 (4.4)	0.798
***Cardiovascular risk factors, n (%)***				
Arterial hypertension	120 (92.3)	78 (91.8)	42 (93.3)	1.000
Prior smoking	19 (14.6)	14 (16.5)	5 (11.1)	0.602
Current smoking	3 (2.3)	2 (2.4)	1 (0.5)	1.000
Diabetes mellitus	33 (25.4)	19 (22.4)	13 (28.9)	0.521
Dyslipidemia	73 (56.2)	46 (54.1)	26 (57.8)	0.715
Positive family history	10 (7.7)	7 (8.2)	3 (6.7)	1.000
***Prior mitral valve interventions***, ***n (%)***				
Prior surgical MVR	1 (0.8)	1 (1.2)	0 (0.00)	1.000
Prior mitral valve TEER	3 (2.3)	2 (2.4)	1 (2.2)	1.000
Prior transcatheter MV annuloplasty (Carillon). *n* (%)	3 (2.3)	0 (0.00)	3 (4.4)	**0**.**04**
***Laboratory parameters* (± standard deviations)**				
GFR, mL/min/1.73 m^2^	50.4 (±20.3)	49.6 (±20.1)	51.9 (±20.5)	0.489
Creatinine, mg/dL	1.5 (±1.05)	1.5 (±1.15)	1.429 (±0.9)	0.720
GOT, U/L	30.7 (±39.59)	24 (±11.5)	43.7 (±64.6)	**0**.**025**
GPT, U/L	32.9 (±80.9)	22.2 (±20.4)	53.4 (±133.7)	**0**.**038**
Albumin, g/dL	3.3 (±0.5)	3.3 (±0.5)	3.4 (±0.5)	0.614
NT-proBNP, ng/L	10 514 (±15 694.23)	9785 (±16 395.2)	11 763.8 (±14 719.4)	0.427
***Echocardiographic parameters* (± standard deviations)**				
LVEF, %	44.28 (±13.34)	49.57 (±11.38)	34.32 (±10.89)	**<0.001**
LV-GLS, %	−15.90 (±6.21)	−18.58 (±5.48)	−11.55 (±4.70)	**<0.001**
LVESD, mm	42.60 (± 12.02)	39.87 (±11.01)	47.81 (±12.26)	**<0.01**
LVEDD, mm	52.18 (±10.65)	50.06 (±10.58)	56.18 (±9.7)	**0**.**002**
LVEDV, mL	160.05 (±62.02)	142.62 (±52.11)	188.41 (±66.82)	**<0.01**
LVEDVI, mL/m^2^	87.18 (±32.20)	7 885 (±27.32)	10 105 (±35.15)	0.456
RV-FAC, %	31.45 (±8.02)	33.79 (±7.29)	27.26 (±7.63)	**<0.001**
TAPSE, mm	19.55 (±5.41)	20.15 (±5.438)	18.62 (±5.29)	0.167
MR severity	2.87 (±0.33)	2.88 (±0.32)	2.83 (±0.35)	0.420
Vena contracta, mm	7.37 (±1.91)	7.22 (±1.93)	7.61 (1.87)	0.284
EROA, mm^2^	44.73 (±21)	44.35 (±2 085)	4 539 (±2 146)	0.693
Disproportional MR, *n* (%)	84 (85.71)	53 (88.33)	31 (81.58)	0.386
Mean mitral valve gradient, mmHg	1.85 (±0.98)	1.86 (±0.94)	1.82 (±1.07)	0.847
TR severity	1.91 (±0.76)	1.9 (±0.76)	1.92 (±0.78)	0.875
Peak gradient TR, mmHg	38.41 (±13.74)	38 (±13.97)	27.2643 (±7.63)	0.654

**Figure 2:**
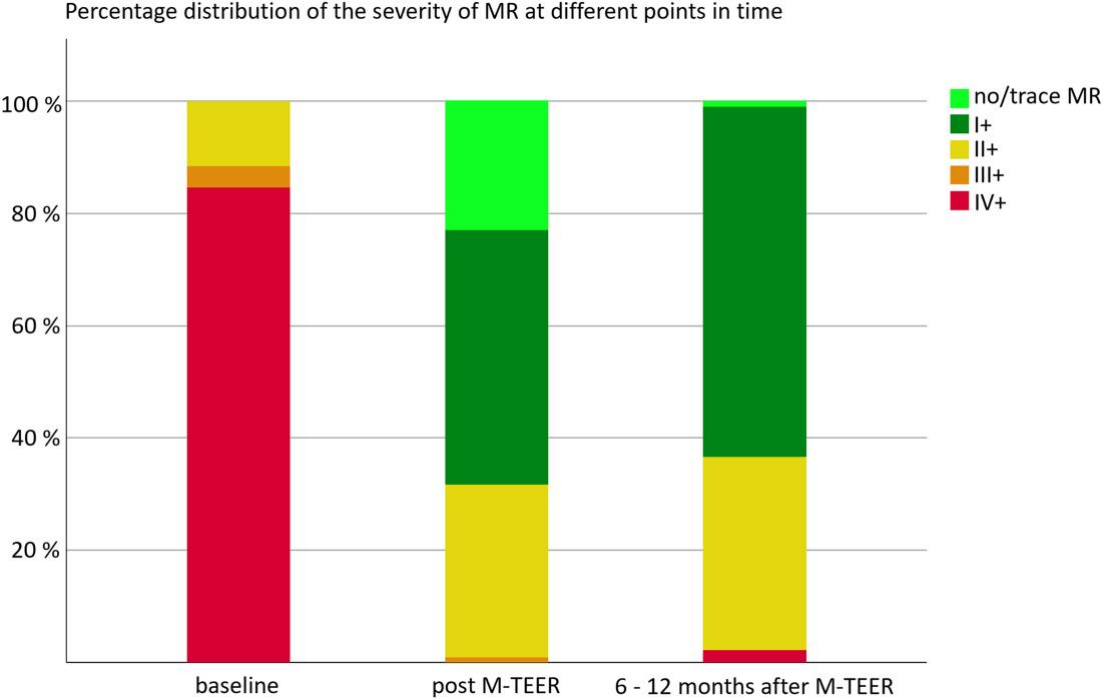
Percentage distribution of the severity of MR at different points in time. Comparison of the severity of mitral regurgitation before M-TEER, immediately after M-TEER, and in the follow-up examination after 6–12 months.

A significant reduction in the severity of MR was observed immediately after the procedure and during follow-up (*P* < 0.001). Before the procedure, only 11.5% of the patients in the cohort had an MR of ≤ 2+, and none had ≤ 1+. Immediately after M-TEER, MR with a degree of ≤ 1+ was present in 68.5% of patients, while MR of ≤ 2+ was present in 99.2%. At the 6–12-month follow-up, 63.5% of patients had MR of ≤ I+, and 97.9% of patients had MR of ≤ II+ (*Figure 2*).

### Change in haemodynamic and echocardiographic parameters

Haemodynamic parameters and their periprocedural changes, compared between patients with primary and secondary MR, are summarized in *Table 2*. A significant relative improvement of 11.37% in CI (*P* < 0.01) was observed, alongside reductions in LA pressure (−36.97%, *P* < 0.01), V-wave (−51.4%, *P* < 0.01), and PCWP (−12.09%, *P* < 0.033). Changes in CI, LA pressure, and V-wave were significant in both the overall cohort and in the subgroup analyses of patients with primary and secondary MR. In patients with secondary MR, the percentage improvement in CI was more pronounced (43.7%; *P* = 0.022).

**Table 2:** Comparison of haemodynamic parameters before and after M-TEER

	Pre-M-TEER	Immediately post-M-TEER	*P*-value
***All patients***			
CI, l/min/m^2^ (*n* = 76)	2.9 (±0.9)	3.2 (±0.1)	**<0**.**01**
v-wave, mmHg (*n* = 73)	23.9 (±13.1)	14.1 (±7.8)	**<0.01**
LA-pressure, mmHg (*n* = 68)	13.6 (±7.3)	9.4 (±5.8)	**<0.01**
PCWP, mmHg (*n* = 84)	12.9 (±7.4)	11.4 (±7.6)	**0.017**
mPAP, mmHg (*n* = 70)	25.7 (±9.0)	24.3 (±10.3)	0.099
***Patients with primary MR***			
CI, l/min/m^2^ (*n* = 52)	3 (±1)	3.3 (±1)	**0.012**
v-wave, mmHg (*n* = 44)	24.1 (±14.7)	14.6 (±7.74)	**<0.01**
LA pressure, mmHg (*n* = 41)	12.7 (±7.5)	8.93 (±5.7)	**<0.001**
PCWP, mmHg (*n* = 57)	11.7 (±6.8)	10.3 (±6.9)	**0.088**
mPAP mean, mmHg (*n* = 50)	24.4 (±8.2)	22.4 (±9.2)	**0.186**
***Patients with secondary MR***			
CI, l/min/m^2^ (*n* = 24)	2.7 (±0.8)	3 (±0.9)	**0.022**
v-wave, mmHg (*n* = 29)	23.5 (±10.3)	13.5 (±7.9)	**<0.001**
LA pressure, mmHg (*n* = 28)	15.1 (±6.9)	10. (±6.)	**<0.001**
PCWP, mmHg (*n* = 27)	15.4 (±1.5)	13.9 (±8.5)	0.118
mPAP mean, mmHg (*n* = 18)	29.4 (±10.5)	30.1 (±11.4)	0.229

*Table 3* summarizes the echocardiographic parameters before and 6–12 months after M-TEER. Overall, we observed a significant reduction in LVEDD by an average of 2.8 mm (−4.0%, *P* = 0.004). Additionally, a significant improvement in RV-FAC of 5.8% (*P* = 0.019) was observed. Furthermore, we noticed a slight trend towards improvement in LV-GLS and LVEF of 3.7% and 1.39%, respectively, though this was not statistically significant. The parameters were also compared between subgroups of patients with primary and secondary MR. In patients with secondary MR, we also observed a modest trend towards improvement in LV-GLS and LVEF of 11.1% and 5.9%, respectively, which was more pronounced than in patients with primary MR but was though not statistically significant. The change in RV-FAC as a measure of right ventricular function was significant only in the overall cohort (*P* = 0.02) and in the subgroup with secondary MR (*P* = 0.01), but not in the group with primary MR.

**Table 3:** Comparison of echocardiographic parameters before and after M-TEER

	Pre-M-TEER	Post-M-TEER (6–12 months)	*P*-value
***All patients***			
LV-GLS, % (*n* = 85)	−15.9 (±6.3)	−16.4 (± 5.8)	0.363
LVEF, % (*n* = 93)	44.9 (±12.93)	45.1 (±12.1)	0.742
LVESD, mm (*n* = 102)	43.3 (±12.4)	40.2 (±10.9)	**0**.**003**
LVEDD, mm (*n* = 103)	52.8 (±11.05)	50 (±0.3)	**0**.**004**
TAPSE, mm (*n* = 62)	19.9 (±5.3)	18.66 (±5.2)	0.088
RV-FAC, % (*n* = 89)	31.5 (±8)	33.6 (±7.3)	**0**.**019**
PG-TR, mmHg (*n* = 78)	39 (±14.7)	35.6 (±13.7)	0.078
Vena contracta, mm (*n* = 57)	7.1 (±1.9)	3.2 (±1.3)	**<0**.**01**
***Patients with primary MR***			
LV-GLS, % (*n* = 52)	−18.6 (±5.5)	−18.5 (±4.5)	0.90
LVEF, % (*n* = 61)	49.8 (±11.2)	49.2 (±9.6)	0.57
LVESD, mm (*n* = 65)	40.6 (±11.4)	37 (±9.7)	**0**.**002**
LVEDD, mm (*n* = 66)	50.5 (±10.9)	47.8 (±9.1)	**0**.**026**
TAPSE, mm (*n* = 38)	20.7 (±5.8)	19 (±5.4)	0.075
RV-FAC, % (*n* = 55)	34.2 (±7)	34.8 (±7.3)	0.508
Peak gradient TR, mmHg (*n* = 53)	38.3 (±14.8)	35.4 (±14.3)	0.21
Vena contracta, mm (*n* = 44)	6.8 (±1.7)	3.2 (±1.1)	**<0**.**01**
***Patients with secondary MR***			
LV-GLS, % (*n* = 33)	−11.6 (±4.9)	−13 (±6.1)	0.087
LVEF, % (*n* = 32)	35.3 (±10.7)	37.3 (±12.5)	0.172
LVESD, mm (*n* = 37)	48.1 (±12.8)	45.9 (±10.9)	0.294
LVEDD, mm (*n* = 37)	56.8 (±10.1)	53.9 (±8.4)	0.068
TAPSE, mm (*n* = 24)	18.6 (±4.1)	18.2 (±5.)	0.681
RV-FAC, mm (*n* = 34)	27.2 (±7.7)	31.6 (±6.9)	**0**.**009**
Peak gradient TR, mmHg (*n* = 25)	40.6 (±14.5)	36.0 (±12.4)	0.211
Vena contracta, mm (*n* = 13)	8.2 (±2.4)	3.2 (±1.9)	**<0**.**01**

### Correlations between haemodynamic and echocardiographic parameters

**Figure 3:**
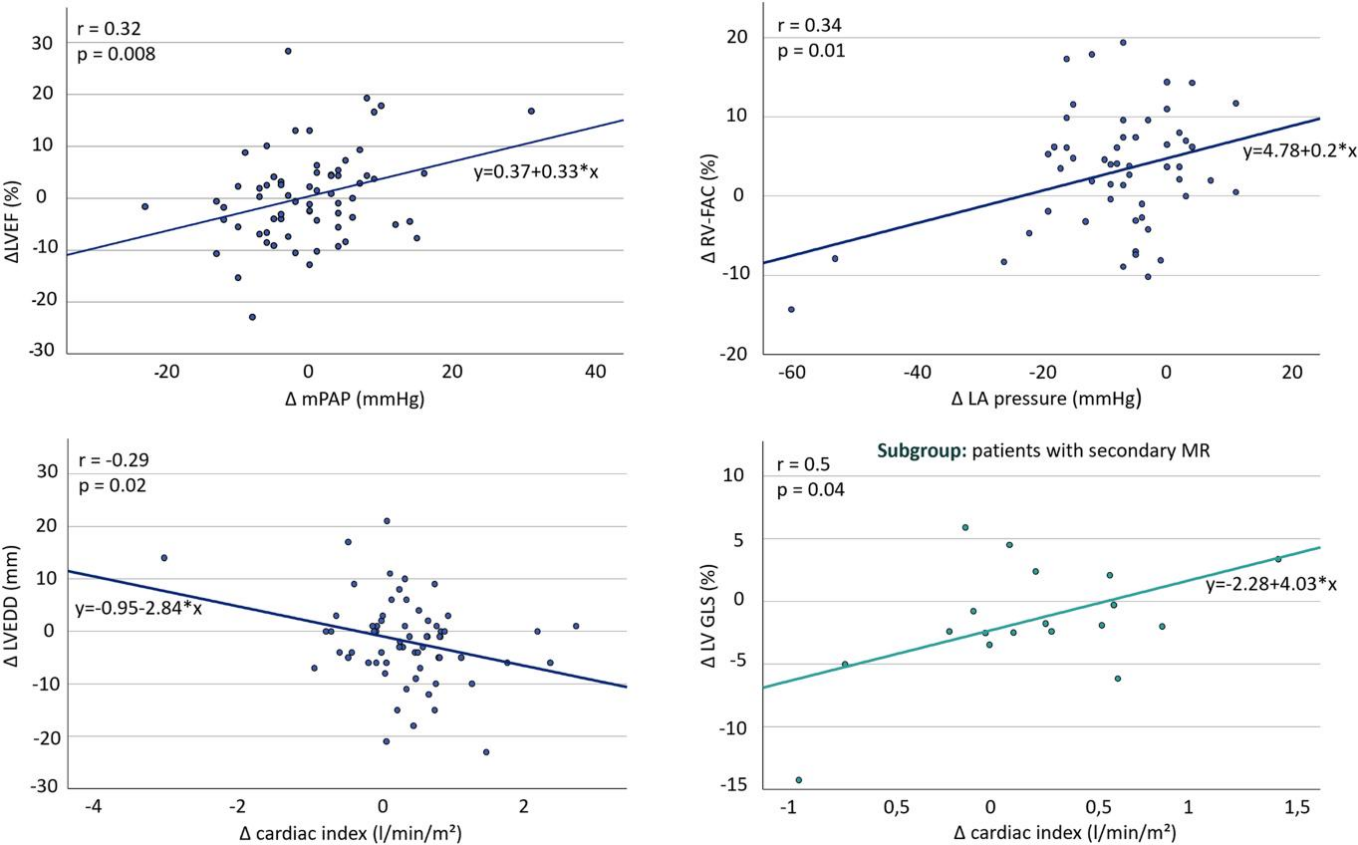
Correlations between haemodynamic and echocardiographic parameters. The correlation between the change in LVEF and the change in mPAP after M-TEER is shown in the top left (*r* = 0.32, *P* = 0.008). The correlation between the change in RV-FAC and the change in left atrial pressure is shown in the top right (*r* = 0.34, *P* = 0.01). The correlation between the change in LVEDD and the change in CI is shown in the bottom left (*r* = 0.29, *P* = 0.02). The correlation between the change in LV-GLS and the change in CI is shown in the bottom right (*r* = 0.5, *P* = 0.04).

Correlation analysis was used to analyse the relationship between periprocedural changes in haemodynamic parameters and the follow-up echocardiographic parameters (*Figure 3*). Significant correlations were demonstrated between a reduction in mPAP and an improvement in LVEF (*r* = 0.32, *P* = 0.008), and between the reduction in LA pressure and an improvement in RV-FAC (*r* = 0.34, *P* = 0.012). When the primary and secondary MR subgroups are considered separately, the following findings emerge: In patients with secondary MR, a significant correlation was demonstrated between improvements in CI and LV-GLS (*r* = 0.5, *P* = 0.04). No other significant correlations were found in this subgroup. In patients with primary MR, the correlations between LVEF and mPAP (*r* = 0.3, *P* = 0.03), as well as that between RV-FAC and LA pressure (*r* = 0.4, *P* = 0.01), are significant. The main results are shown in *Figure 3*. The detailed results of all correlation analyses can be found in Supplementary data online, *Table S1*.

### Clinical and functional outcomes

**Figure 4:**
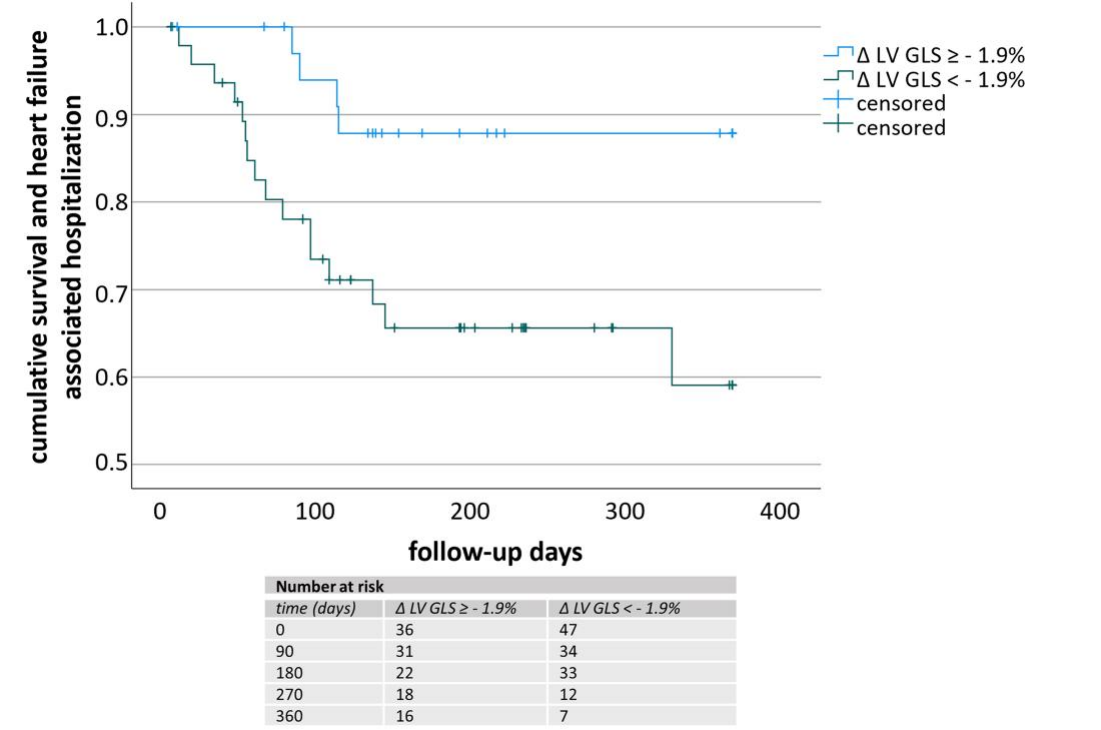
Kaplan–Meier survival estimates for the occurrence of hospitalization and death. This plot shows the survival functions for hospitalizations due to heart failure and for deaths, separated according to whether or not there was an improvement in LVGLS of at least 1.9% after the intervention. The number of patients at risk is shown below the *x*-axis in 90-day intervals.

The clinical and functional outcomes are summarized in Supplementary data online, *Table S1*. A significant improvement in NYHA class was observed at follow-up. Regarding our primary combined endpoint (all-cause mortality and heart failure-associated hospitalization), we demonstrated lower event rates occurring (*P* = 0.01) in patients with an improvement in LV-GLS of at least −1.9% (*Figure 4*). This threshold was determined using receiver operating characteristic analysis.

### Success according to MVARC criteria

Technical, device, procedural, and patient success were assessed as described above, with results summarized in *Table 4*. Technical success was achieved in 95.4% of patients, and device success immediately after intervention was observed in 93.1%. At follow-up, ∼83% of patients maintained device success. Procedural success at follow-up was achieved in ∼67% of patients, while patient success was reached in ∼60% of the cohort.

**Table 4:** Success of intervention based on the official MVARC criteria

	*n*	Entire cohort	*n*	Primary MR	*n*	Secondary MR	*P*-value
Technical success, *n* (%)	130	124 (95.4)	85	82 (96.5)	45	42 (93.3)	0.416
Procedural success in FU, *n* (%)	110	74 (67.3)	69	44 (63.8)	41	30 (73.2)	0.401
Patient success in FU, *n* (%)	106	63 (59.4)	66	37 (56.1)	40	26 (65)	0.418
Device success directly after intervention, *n* (%)	130	121 (93.1)	85	78 (91.78)	45	43 (95.6)	0.201
Device success in FU, *n* (%)	112	93 (83.04)	71	57 (80.3)	41	36 (87.8)	0.302
Optimal device success directly after intervention, *n* (%)	130	37 (28.5)	85	20 (23.5)	45	17 (37.8)	0.201
Acceptable device success directly after intervention, *n* (%)	130	84 (64.6)	85	58 (68.2)	45	26 (57.8)	0.201
Optimal device success in FU, *n* (%)	112	11 (9.8)	71	5 (7)	41	6 (8.5)	0.302
Acceptable device success in FU, *n* (%)	112	82 (73.21)	71	52 (73.2)	41	30 (73.2)	0.302

### Determinants of outcome

A multivariate Cox regression analysis was performed to identify potential predictors of patient survival after M-TEER. Of all the analysed predictors, an increased post-interventional V-wave (*P* = 0.05, 95% CI 0.99–1.16) and a lack of improvement of LV-GLS (*P* = 0.008, 95% CI 0.94–0.99) were found to be significantly associated with an increased mortality rate within the first year after M-TEER. Residual MR after M-TEER, time to follow-up echocardiography, and MR aetiology were not independently associated with clinical outcome. The results of the multivariate analysis are summarized in *Table 5*.

**Table 5:** Results of the Cox regression to analyse the risk factors for composite endpoint of all-cause mortality and heart failure–related hospitalization in the first year after M-TEER

Variable	Hazard ratio (HR)	95% CI	*P*-value
MR immediately after M-TEER	0.88	0.41–1.88	0.73
Primary or secondary MR	0.64	0.81–2.29	0.5
Days between M-TEER and follow-up	1	0.99–1.01	0.83
Proportional or disproportional MR	1.59	0.19–12.78	0.66
V-wave post-M-TEER	1.08	0.99–1.16	**0**.**05**
Relative improvement of LV-GLS	0.97	0.94–0.99	**0**.**008**

In multivariable linear regression analysis, a greater periprocedural increase in CI (*P* = 0.036) was independently associated with improvement in LV-GLS at follow-up, after adjustment for MR aetiology, baseline LV-GLS, and changes in pulmonary artery pressure. Baseline LV-GLS was also strongly associated with subsequent improvement (*P* < 0.001). The overall model demonstrated a moderate explanatory power (*R*^2^ = 0.40; adjusted *R*^2^ = 0.34). The results are summarized in *Table 6*.

**Table 6:** Multivariable linear regression analysis for predictors of improvement in LV-GLS at follow-up

Covariates	Standardized *β*	95% CI	*P*-value
Cardiac index change	−0.27	−24.9 (−48.2 to −1.7)	**0**.**036**
Baseline LV-GLS	0.72	7.80 (4.47 to 11.13)	**<0**.**001**
MR etiology (primary vs. secondary)	−0.24	−36.2 (−80.9 to 8.5)	0.110
Change in pulmonary artery pressure (ΔmPAP)	0.17	1.60 (−0.75 to 3.95)	0.176

**Model statistics:**  *R*^2^ = 0.399.

Adjusted *R*^2^ = 0.34.

*n* = 85.

## Discussion

This study analysed peri- and post-procedural haemodynamic and echocardiographic changes, as well as their relationship with clinical outcomes, in 130 patients with severe MR who underwent M-TEER with the PASCAL system (Edwards Lifesciences, CA, USA). The main findings are summarized below.

### Immediate haemodynamic effects of M-TEER and subsequent echocardiographic findings at follow-up

First, M-TEER resulted in an immediate improvement in haemodynamic parameters, an increase in CI, and a reduction in LA pressure and V-wave in patients with both primary and secondary MR. These findings are consistent with those observed in MR treatment with MitraClip system (Abbott).^12^

Secondly, a significant reduction of LVEDD in the follow-up examination was observed. This suggests modest changes in LV geometry after M-TEER. In addition, a slight improvement in LV-GLS and LVEF was observed, though not significant, and more pronounced in patients with secondary MR. This may be explained by the already impaired baseline LV-GLS in this subgroup. In contrast, patients with primary MR—who comprised the majority of the cohort—typically exhibited preserved baseline LV-GLS, limiting the potential for detectable improvement. Accordingly, more pronounced changes in LV-GLS might be expected in cohorts restricted to secondary MR or with markedly reduced baseline LV-GLS.

Thirdly, the significant improvement in RV-FAC in the entire cohort can most likely be explained by a decreased LA pressure, which could indicate a decrease in right ventricular afterload. Correlation analysis also showed that a reduction in mPAP was significantly correlated with an improvement in LV function, and a significant correlation was observed between a reduction in LA pressure and an improvement in RV-FAC.

Last but not least, it was found that the relative improvement in CI immediately following M-TEER was independently associated with subsequent improvement in LV-GLS in follow-up echocardiography. This finding suggests that the acute optimization of haemodynamics achieved by M-TEER might translate into favourable myocardial remodelling over time. Importantly, this association remained robust after adjustment for clinically relevant confounders, including MR aetiology, baseline LV-GLS, and changes in pulmonary artery pressure, supporting a potential mechanistic link between improved forward flow and reverse remodelling. However, it should be noted that the overall change in LV-GLS within the cohort did not reach statistical significance, indicating that while individual haemodynamic responses may predict remodelling, the effect at the population level remains modest.

### Clinical effects of M-TEER-induced haemodynamic improvement and resulting cardiac remodelling

The primary endpoint of the study was a composite of mortality and heart failure-associated hospitalization. Kaplan–Meier analysis suggests that an improvement in LV-GLS of at least −1.9% was associated with significantly lower rates of mortality and hospitalization.

An increased post-interventional V-wave was significantly associated with our composite endpoint of mortality and heart failure-associated hospitalization, even after adjusting for residual MR severity, which itself did not emerge as a significant predictor. This suggests that less reduction in LA pressure might be considered as a marker for a worse procedural outcome, which in turn could be associated with a higher mortality. Overall, this supports our interpretation that acute invasive haemodynamic changes are not merely surrogate markers for MR reduction, but might reflect a more comprehensive haemodynamic response with independent prognostic relevance.

Additionally, a study that used invasive pressure–volume loops measurements showed that patients with elevated V-wave pressure above 20 mmHg have impaired LV diastolic function and increased LA stiffness, which could also be the case for our patients.^22^

### Prognostic impact of disproportionate MR

The presence of disproportionate MR in patients with functional mitral regurgitation (FMR) was also investigated to determine its impact on our primary endpoint of heart failure-related hospitalizations and mortality. In this real-world cohort, disproportionate MR was not significantly associated with heart failure-related hospitalizations and mortality; larger studies are needed to validate these findings. In the COAPT study, which demonstrated a significant reduction in 2-year mortality with the MitraClip® system, patients generally had disproportionate MR. Conversely, the MITRA-FR study, which yielded neutral results, included patients with more proportionate MR, where MR may have been a bystander in the context of severe LV dysfunction.^23,24^ Compared to these cohorts, our FMR patients had slightly better ventricular function, with an average LVEF of 34.3% (vs. 31% in COAPT, 33% in MITRA-FR, and 31% in RESHAPE HF-2).^25^ The average LVEDV in our FMR group was 188 mL, lower than in COAPT (193 mL), MITRA-FR (250 mL), and RESHAPE HF-2 (211 mL). The mean LVEDV index was 101.1 mL/m^2^, lower than MITRA-FR (135 mL/m^2^) and equal to COAPT (101 mL/m^2^).^26,27^ Notably, the average EROA in our FMR group was 45.4 mm^2^, higher than in COAPT (41 mm^2^), MITRA-FR (31 mm^2^), and RESHAPE HF-2 (25 mm^2^), indicating that most of our FMR patients had disproportionate MR.^11,28,29^

### MVARC-defined success

To demonstrate the effectiveness of the PASCAL® implant system, this study also evaluated technical, device-related, procedural, and patient success according to the MVARC criteria.^21^ Technical success was achieved in 95.4% of all the patients, consistent with previously reported rates of 90–95.1% for PASCAL® and MitraClip® systems, indicating a low rate of periprocedural complications despite a highly comorbid population.^30^ Device success as measured immediately after the intervention was achieved in 93.1% of patients, decreasing slightly to 83% during the 1-year follow-up. The most common reasons for this were an increased mitral valve gradient and recurrent worsening of MR. Comparable discharge device success rates of 94.8% have been reported in the MiCLASP registry, without significant differences between primary MR and FMR; follow-up data on device success were not available here.^31^ In contrast, Geis *et al.* reported lower device success rates at discharge for MitraClip® (89.1%) and slightly lower rates for the PASCAL® implant system (90.2%).^30^

Patient success was observed in around 60%, which was rather low. This was probably due to relatively high hospitalization rates after M-TEER in our severely ill patient population, emphasizing the importance of patient selection. Long-term MVARC endpoints remain inconsistently reported across studies, limiting comparability beyond the periprocedural period. Nevertheless, the reduction in MR in the follow-up as well as the rates of heart failure hospitalization and mortality can be compared: At 6–12 months, MR ≤2 + was observed in 97.9% of patients. Quite similar results were reported after 1 year in the MiCLASP registry (MR ≤2+: 97.9%)^31^ and EXPAND (MR ≤2+: 97.5%).^32^ The combined rate of heart failure hospitalization and mortality at 1 year was 28.5% in our cohort, compared with 23.6% in MiCLASP.^31^ In EXPAND, 1-year heart failure hospitalization and mortality rates were 18.9% and 14.9%, respectively, without a combined endpoint.^32^ These broadly comparable event rates may be explained by similarities in baseline characteristics across cohorts, including mean age (79.4 vs. 77.1 vs. 77.3 years) and proportion of FMR (34.6% vs. 59.2% vs. 39.7%).^31,32^

Overall, the high technical and device success rates confirm that M-TEER with the PASCAL® implant system is a safe procedure that achieves good post-interventional results with sustained MR reduction after 6–12 months.

## Study limitations

One main limitation of this study is that echocardiographic measurements were not performed by an independent core laboratory; however, all echocardiograms were reviewed and analysed by two independent physicians. In this context, it should also be noted that the echocardiographic follow-up examinations were spread out over a period of 6–12 months following M-TEER; this was due to the retrospective nature of the data collection, which could have introduced bias into the results. To investigate this limitation, the number of days between M-TEER and the echocardiographic follow-up was included in our Cox regression analysis and was not found to be a significant predictor of outcome. The limited sample size (*n* = 130) and low event rate should be considered when interpreting the results, particularly given the increased risk of overfitting in multivariable models. Frame rates were not systematically recorded due to the retrospective study design and use of different ultrasound systems, which may have influenced strain measurements.

It should also be pointed out that, although efforts were made to standardize sedation levels, blood pressure, and heart rate during measurements, some variability may have influenced the results. Post-procedural assessment of LV-GLS was not feasible in all patients due to limited echocardiographic image quality, and periprocedural haemodynamic data were not available for every patient. Another important limitation worth mentioning is the fact that the ROC-derived LV-GLS cutoff is data-driven, which increases the risk of overfitting as well as of overestimating its prognostic value. Finally, the follow-up period was restricted to 1 year, precluding assessment of long-term outcomes.

## Conclusion

In patients with severe symptomatic MR, M-TEER using the PASCAL® system resulted in an immediate improvement of several haemodynamic parameters, with possible reverse cardiac remodelling and corresponding clinical benefit.

## Supplementary Material

qyag106_Supplementary_Data

## Data Availability

The data underlying this article will be shared on reasonable request to the corresponding author.

## Lead Author Biography

Tobias Geisler is Professor of Cardiology and Vice Head of the Department of Cardiology at the University Hospital Tübingen, Germany. After his training in cardiology, he worked as a senior research fellow at the Royal Brompton Hospital/Imperial College in London. He is an interventional cardiologist and leads the cardiovascular clinical research unit at the University Hospital Tübingen. He was a member of the steering board of the Collaborative Research Consortium TR240 ‘Platelets – Molecular, cellular and systemic functions in health and disease’ funded by the German Research Foundation. He has (co-) authored more than 300 peer-reviewed articles with over 10 000 citations. His H-index is 52 (Scopus). His main research interests include platelet biology, pharmacogenomics of antiplatelet and antithrombotic therapies, stroke, translational medicine, clinical trials, and structural heart interventions, including TAVR, TEER, TMVR, and TTVR. He is the section editor of Thrombosis and Haemostasis and J Thrombosis and Thrombolysis and was chair of the Working Group on Thrombosis by the German Cardiac Society and secretary and treasurer of the ESC Working Group on Thrombosis.
