# Supplementary material for: Cardiac remodelling and clinical outcomes after mitral edge-to-edge repair with the PASCAL® system
Source: Eur Heart J Imaging Methods Pract. 2026 Jun 12;4(3):qyag106. doi: 10.1093/ehjimp/qyag106 (PMC13326949; doi:10.1093/ehjimp/qyag106)
Supplement: qyag106_Supplementary_Data [file qyag106_supplementary_data.docx]

**Cardiac remodeling and clinical outcomes after mitral edge-to-edge repair with the PASCAL**® **system**

Mareike Bladt^1^, Marius Keller^2^, Meinrad Gawaz^1^, Juergen Schreieck^3^, Andreas Goldschmied^1^, Makoto Amaki^4^, Takashi Matsumoto^5^, Izumo Masaki^6^, Shingo Kuwata^6^, Shunsuke Kubo^7^, Kentaro Hayashida^8^, Firas Zahr^9^, Felix Kreidel^10^, Fabien Praz^11^, Didier Tchétché ^12^, Harry Magunia^2^, Monika Zdanyte^1^, Tobias Geisler^1^

^1^ Department of Cardiology and Angiology, University Hospital Tübingen, Otfried-Müller-Straße 10, 72076 Tübingen, Germany

^2^ Department of Anaesthesiology and Intensive Care Medicine, University Hospital Tübingen, Hoppe-Seyler-Str. 3, 72076, Tübingen, Germany

^3^ Department of Cardiology, Medius Kliniken gGmbH medius Klinik, Ostfildern-Ruit, Germany

**^4^** Department of Heart Failure and Transplant Division of Heart Failure National Cerebral and Cardiovascular Center, 6-1 Kishibeshinmachi Suita, Osaka 564-8565, Japan

^5^ Department of Cardiology and Cath Laboratories, Shonan Kamakura General Hospital, Japan

^6^ Division of Cardiology, Department of Internal Medicine, St. Marianna University School of Medicine, Kawasaki, Japan

^7^ Department of Cardiology Kurashiki Central Hospital Kurashiki Japan

^8^ Department of Cardiology, Keio University School of Medicine, 35 Shinanomachi, Shinjyuku-ku, Tokyo, 160-8582, Japan

^9^ Division of Cardiovascular Medicine, Knight Cardiovascular Institute, Oregon Health & Science University, Portland, Oregon, USA

^10^ Department of Internal Medicine III, Cardiology and Critical Care, University Hospital Schleswig-Holstein, Campus Kiel, Arnold-Heller-Straße 3, 24105 Kiel, Germany

^11^ Department of Cardiology, Inselspital, Bern University Hospital, Freiburgstrasse 18, 3010 Bern, Switzerland

^12^ Clinique Pasteur, Toulouse, France.

**Correspondence:**

Prof. Tobias Geisler

Department of Cardiology and Angiology, University Hospital Tübingen, Otfried-Müller-Straße 10, 72076 Tübingen, Germany

E-Mail: tobias.geisler@med.uni-tuebingen.de

Telephone: +4970712984405

**Supplementary material**

Tables:

***Table S1.*** Tilte: Correlation between echocardiographic and hemodynamic parameters

| **All patients** | | | |
| --- | --- | --- | --- |
| *Δ* ***LV-GLS*** | | | |
| **Variable 1** | **Variable 2** | **Correlation coefficient** | **p value** |
| Δ LV-GLS | Δ CI | 0,012 | 0,933 |
| Δ LV-GLS | Δ LA pressure | -0,040 | 0,781 |
| Δ LV-GLS | Δ PCWP | 0,134 | 0,333 |
| Δ LV-GLS | Δ mPAP | -0,148 | 0,262 |
| *Δ* ***LVEF*** | | | |
| **Variable 1** | **Variable 2** | **Correlation coefficient** | **p value** |
| Δ LVEF | Δ CI | -0,026 | 0,848 |
| Δ LVEF | Δ LA pressure | 0,128 | 0,344 |
| Δ LVEF | Δ PCWP | -0,092 | 0,487 |
| Δ LVEF | Δ mPAP | 0,321 | **0,008** |
| *Δ* ***RV-FAC*** | | | |
| **Variable 1** | **Variable 2** | **Correlation coefficient** | **p value** |
| Δ RV-FAC | Δ CI | 0,196 | 0,152 |
| Δ RV-FAC | Δ LA pressure | 0,344 | **0,012** |
| Δ RV-FAC | Δ PCWP | -0,017 | 0,898 |
| Δ RV-FAC | Δ mPAP | 0,040 | 0,753 |
| *Δ* ***LVEDD*** | | | |
| **Variable 1** | **Variable 2** | **Correlation coefficient** | **p value** |
| Δ LVEDD | Δ CI | -0,287 | **0,022** |
| Δ LVEDD | Δ LA pressure | 0,083 | 0,513 |
| Δ LVEDD | Δ PCWP | -0,158 | 0,204 |
| Δ LVEDD | Δ mPAP | 0,042 | 0,719 |
| **primary MR** | | | |
| *Δ* ***LV-GLS*** | | | |
| **Variable 1** | **Variable 2** | **Correlation coefficient** | **p value** |
| Δ LV-GLS | Δ CI | -0,139 | 0,448 |
| Δ LV-GLS | Δ LA pressure | -0,072 | 0,707 |
| Δ LV-GLS | Δ PCWP | 0,140 | 0,422 |
| Δ LV-GLS | Δ mPAP | -0,049 | 0,765 |
| *Δ* ***LVEF*** | | | |
| **Variable 1** | **Variable 2** | **Correlation coefficient** | **p value** |
| Δ LVEF Simpson | Δ CI | 0,001 | 0,996 |
| Δ LVEF Simpson | Δ LA pressure | 0,144 | 0,402 |
| Δ LVEF Simpson | Δ PCWP | 0,015 | 0,924 |
| Δ LVEF Simpson | Δ mPAP | 0,312 | **0,033** |
| *Δ* ***RV-FAC*** | | | |
| **Variable 1** | **Variable 2** | **Correlation coefficient** | **p value** |
| Δ RV-FAC | Δ CI | 0,244 | 0,152 |
| Δ RV-FAC | Δ LA pressure | 0,437 | **0,014** |
| Δ RV-FAC | Δ PCWP | 0,181 | 0,290 |
| Δ RV-FAC | Δ mPAP | -0,057 | 0,715 |
| *Δ* ***LVEDD*** | | | |
| **Variable 1** | **Variable 2** | **Correlation coefficient** | **p value** |
| Δ LVEDD | Δ CI | -0,248 | 0,109 |
| Δ LVEDD | Δ LA pressure | 0,077 | 0,639 |
| Δ LVEDD | Δ PCWP | -0,108 | 0,487 |
| Δ LVEDD | Δ mPAP | 0,098 | 0,485 |
| **Secondary MR** | | | |
| *Δ* ***LV-GLS*** | | | |
| **Variable 1** | **Variable 2** | **Correlation coefficient** | **p value** |
| Δ LV-GLS | Δ CI | 0,495 | **0,037** |
| Δ LV-GLS | Δ LA pressure | 0,129 | 0,567 |
| Δ LV-GLS | Δ PCWP | 0,172 | 0,481 |
| Δ LV-GLS | Δ mPAP | -0,326 | 0,160 |
| *Δ* ***LVEF*** | | | |
| **Variable 1** | **Variable 2** | **Correlation coefficient** | **p value** |
| Δ LVEF Simpson | Δ CI | -0,077 | 0,762 |
| Δ LVEF Simpson | Δ LA pressure | 0,087 | 0,709 |
| Δ LVEF Simpson | Δ PCWP | -0,242 | 0,318 |
| Δ LVEF Simpson | Δ mPAP | 0,351 | 0,129 |
| *Δ* ***RV-FAC*** | | | |
| **Variable 1** | **Variable 2** | **Correlation coefficient** | **p value** |
| Δ RV-FAC | Δ CI | 0,153 | 0,531 |
| Δ RV-FAC | Δ LA pressure | 0,126 | 0,577 |
| Δ RV-FAC | Δ PCWP | -0,192 | 0,417 |
| Δ RV-FAC | Δ mPAP | 0,207 | 0,367 |
| *Δ* ***LVEDD*** | | | |
| **Variable 1** | **Variable 2** | **Correlation coefficient** | **p value** |
| Δ LVEDD | Δ CI | -0,440 | 0,046 |
| Δ LVEDD | Δ LA pressure | 0,118 | 0,582 |
| Δ LVEDD | Δ PCWP | -0,304 | 0,169 |
| Δ LVEDD | Δ mPAP | -0,077 | 0,721 |

Abbreviations: CI – Cardiac Index, LA pressure – left atrial pressure, PCWP – pulmonary capillary wedge pressure, mPAP – mean pulmonary arterial pressure, LV-GLS – left ventricular global longitudinal strain, LVEF – left ventricular ejection fraction, LVEF Simpson – left ventricular ejection fraction measured by Simpson’s method, RV-FAC – right ventricular fractional area change, LVEDD – left ventricular end-diastolic diameter, MR – mitral regurgitation

***Table S2.*** Tilte: Clinical outcome after M-TEER

|  | **n** | **All patients** | **n** | **primary MR** | | **n** | **secondary MR** | **p value** |
| --- | --- | --- | --- | --- | --- | --- | --- | --- |
| All-cause-mortality 1 year, n (%) | 130 | 18 (13.9) | 85 | 10 (11.8) | | 45 | 8 (17.8) | 0.425 |
| Hospitalisation due to heart failure 1 year, n (%) | 130 | 24 (18.5) | 85 | 14 (16.5) | | 45 | 10 (22.2) | 0.479 |
| ***Development of functional status (NYHA class)*** | | | | | | | | |
|  | **n** | **pre M-TEER** | | | **post M-TEER (after 6-12 months)** | | | **p value** |
| All patients, mean (± SD) | 100 | 3.1 (± 0.4) | | | 1.9 (± 0.7) | | | **< 0.01** |
| Patients with primary MR, mean (± SD) | 64 | 3. (± 0.3) | | | 1.9 (± 0.7) | | | **< 0.01** |
| Patients with secondary MR, mean (± SD) | 36 | 3.2 (± 0.5) | | | 1.9 (± 0.7) | | | **< 0.01** |

Figures

**Figure S1.** Title: Multivariable predictors of LV-GLS improvement at follow-up


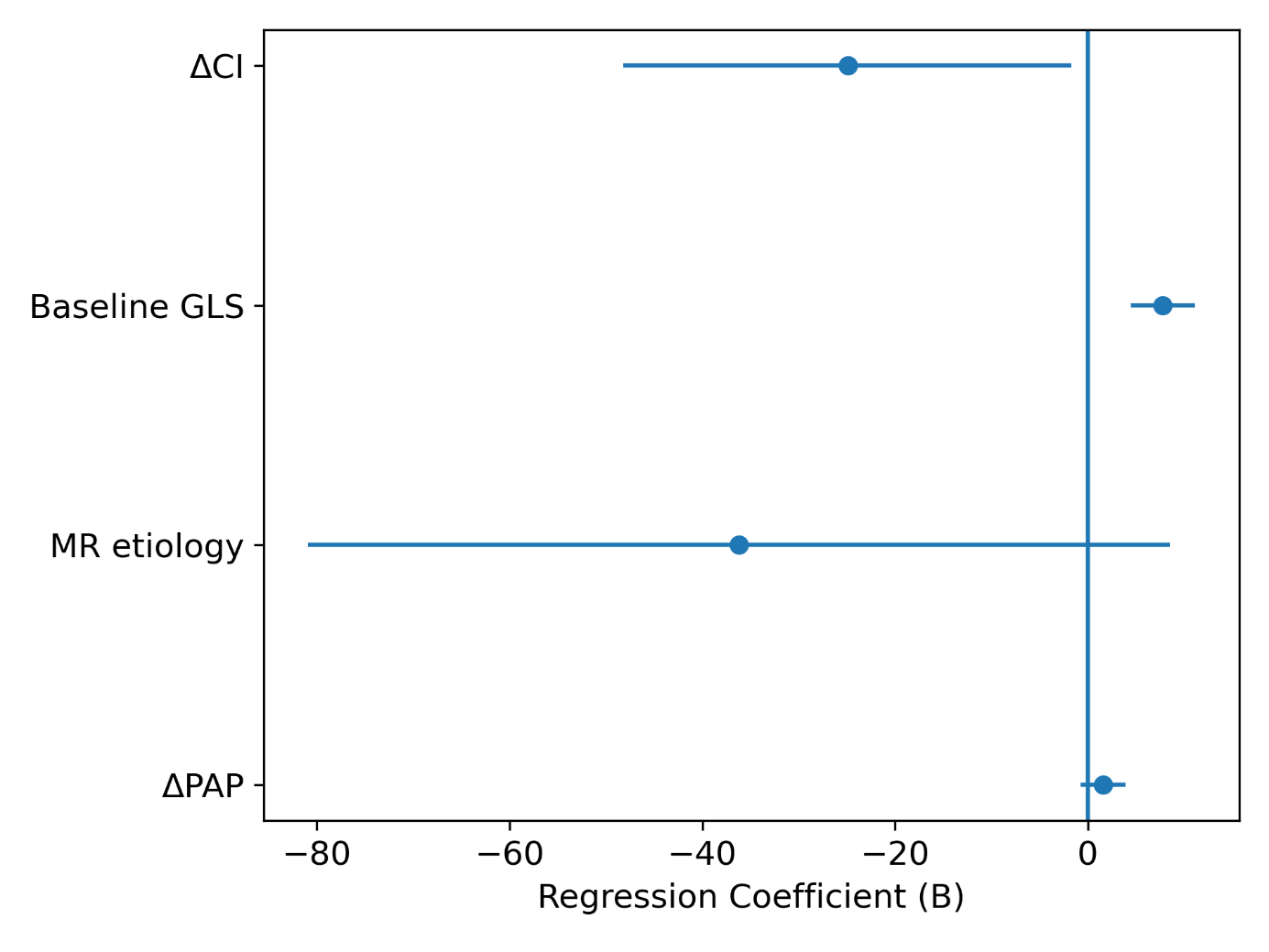


Legend: Forest plot showing regression coefficients (B) with 95% confidence intervals derived from the multivariable linear regression model. A greater periprocedural increase in cardiac index (ΔCI) was independently associated with improvement in LV-GLS at follow-up. Baseline LV-GLS was also strongly associated with subsequent remodeling, while MR etiology and changes in pulmonary artery pressure were not significant predictors.

Abbreviations: CI: cardiac index; GLS: global longitudinal strain; MR: mitral regurgitation; PAP: pulmonary artery pressure.
